# Efficacy of Revolution® Plus (selamectin plus sarolaner) against *Amblyomma americanum* (lone star ticks) in cats

**DOI:** 10.1186/s13071-025-06962-1

**Published:** 2025-08-01

**Authors:** Susan Holzmer, Adriano F. Vatta, Sara Chapin, Vickie King, Jamie A. E. Myers

**Affiliations:** 1 Zoetis, Veterinary Medicine Research and Development, Global Clinical Development, Statistics and Data Management, Kalamazoo, MI USA; 2 Zoetis, Veterinary Medicine Research and Development, GT Global Clinical Development, Chapel Hill, NC USA

**Keywords:** *Amblyomma americanum*, Cat, Isoxazoline, Lone star tick, Revolution® Plus, Sarolaner, Selamectin, Topical combination

## Abstract

**Background:**

The efficacy of a topical combination product, Revolution® Plus, containing selamectin and sarolaner was evaluated for efficacy against *Amblyomma americanum* ticks in cats.

**Methods:**

Four laboratory dose confirmation efficacy studies were conducted in different geographical regions of the USA using three distinct US isolates of *A. americanum*. In each study, ten domestic short-hair cats were randomly allocated to each treatment group based on pretreatment host-suitability tick counts. Cats were infested with approximately 50 unfed adult ticks on day −2. On day 0, cats received either a topical placebo or a single topical dose of Revolution® Plus at the minimum dosage of 6.0 mg/kg selamectin + 1.0 mg/kg sarolaner. Cats were re-infested with approximately 50 unfed adult ticks each on days 5, 12, 19, 26, and 33. Tick counts (comprising live attached and live free ticks) were conducted at 72 h post-treatment and after each subsequent weekly re-infestation.

**Results:**

No treatment-related adverse reactions occurred in any study. In all studies, placebo-treated cats maintained adequate infestations for the entire study duration, and cats treated with Revolution® Plus had significantly lower (*P* < 0.0001) least squares mean live (attached and free) tick counts than placebo-treated cats at all time points for at least 1 month. Revolution® Plus provided a mean of > 90% effectiveness based on least squares means 72 h after treatment of existing infestations, and maintained > 99.1% effectiveness 72 h after re-infestation through at least day 29.

**Conclusions:**

A single dose of Revolution® Plus administered topically at the minimum label dose of 6.0 mg/kg selamectin + 1.0 mg/kg sarolaner provided treatment and control of *A. americanum* on cats for at least 1 month.

**Supplementary information:**

The online version contains supplementary material available at 10.1186/s13071-025-06962-1.

## Background

*Amblyomma americanum*, the lone star tick, has a distribution in the USA that extends from the Gulf Coast northwards into the northern Mid-West, eastwards to the South Atlantic states, and northeastwards as far north as the Canadian border in some areas [1]. Cats infested with these ticks can experience local irritation at the site of bite wounds and generalized hypersensitivity reactions, with alopecia and anemia developing in cases of heavy infestations [2–4]. *Amblyomma americanum* is a known vector of several protozoal, bacterial, and viral organisms [5–7], including *Francisella tularensis* and *Cytauxzoon felis* [8, 9].

The lone star tick is an ixodid (hard) tick species with multiple life stages (egg, larva, nymph, and adult); larvae, nymphs, and adults typically feed on different hosts [3]. These ticks have a high reproductive capacity and appropriate environmental conditions can promote the presence of large numbers of all stages, albeit during different seasons of the year [2–4]. After questing to find a suitable host, ticks will attach to the animal and begin feeding. There is an important window of opportunity to prevent tick-borne disease transmission because there is a delay between tick attachment and transfer of pathogens. This time period differs between pathogens, but killing ticks before they transmit pathogens will prevent disease [10]. Treatment with Revolution® Plus has been shown to interrupt the life cycle of several tick species by killing the adult ticks, potentially interrupting the transmission of the pathogens the ticks harbor, and hence the spread of disease [11].

Revolution® Plus is a commercial product for use in cats in the USA and multiple other countries (as Revolution® Plus or Stronghold Plus) and contains sarolaner, an isoxazoline ectoparasiticide, as well as selamectin, a macrocyclic lactone endectocide. The product provides broad-spectrum efficacy against a range of parasites, including the black-legged tick (*Ixodes scapularis*), the American dog tick (*Dermacentor variabilis*), the Gulf Coast tick (*Amblyomma maculatum*), and the lone star tick (*A. americanum*) [11, 13]. Herein, we report on the studies conducted to confirm the efficacy of Revolution® Plus against *A. americanum*.

## Methods

Four laboratory studies were conducted to evaluate Revolution® Plus against *A. americanum*. Ticks were obtained from three separate tick colonies so that three different US strains were used for efficacy evaluation (Table 1). Study protocols were approved by the institutional animal care and use committees at each of the study sites. All studies were conducted in accordance with the CVM Guidance for Industry no. 85, Good Clinical Practice [12], and the World Association for the Advancement of Veterinary Parasitology (WAAVP) guidelines for evaluating the efficacy of parasiticides for the treatment, prevention, and control of flea and tick infestations on dogs and cats [14]. In addition, masking was accomplished by separation of functions of study personnel. Dedicated personnel at each site were responsible for the placement of cats in their allotted pens and for their treatment but did not conduct any other observations for each study. All personnel involved in making and recording assessments of safety and efficacy were unaware of treatment assignments.

**Table 1:** Least squares mean tick live (attached and free) counts and percent efficacy relative to placebo following a single topical treatment with Revolution® Plus for cats with existing *Amblyomma americanum* infestations and after subsequent weekly re-infestations

Study	Tick strain	Day^e^	Placebo^f^		Revolution® Plus^f^		% Efficacy	Test statistic	*P*-value
			Mean	Range	Mean	Range			
1	Ecto Services^a^	3	30.2	19–41	3.1	0–21	89.7	9.15	< 0.0001
		8	30.2	10–41	0.0	0–0	100.0	10.20	< 0.0001
		15	28.6	12–40	0.0	0–0	100.0	9.66	< 0.0001
		22	29.0	17–40	0.1	0–1	99.7	9.76	< 0.0001
		29	31.5	19–45	0.0	0–0	100.0	10.64	< 0.0001
		36	27.4	14–50	3.1	0–17	88.7	8.21	< 0.0001
2	Bertek^b^	3	22.5	13–33	1.5	0–15	93.3	8.84	< 0.0001
		8	26.8	16–41	0.0	0–0	100.0	11.28	< 0.0001
		15	23.4	14–34	0.0	0–0	100.0	9.85	< 0.0001
		22	22.6	13–37	0.2	0–1	99.1	9.43	< 0.0001
		29	20.5	13–29	0.0	0–0	100.0	8.63	< 0.0001
		36^g^	17.4	5–40	0.2	0–2	98.9	7.20	< 0.0001
3	Oklahoma State University^c^	3	33.0	12–49	3.8	0–0	88.5	8.91	< 0.0001
		8	35.4	26–47	0.0	0–0	100.0	10.80	< 0.0001
		15	31.4	4–51	0.0	0–0	100.0	9.58	< 0.0001
		22	36.0	17–46	0.0	0–0	100.0	10.99	< 0.0001
		29	36.4	21–48	0.0	0–0	100.0	11.11	< 0.0001
		36	26.9	11–47	0.0	0–0	100.0	8.21	< 0.0001
4	Ecto Services^d^	3	18.2	11–23	0.3	0–2	98.4	11.09	< 0.0001
		8	25.3	18–33	0.0	0–0	100.0	15.68	< 0.0001
		15	26.6	19–38	0.0	0–0	100.0	16.49	< 0.0001
		22	31.5	22–41	0.1	0–1	99.7	19.46	< 0.0001
		29	32.2	23–40	0.1	0–1	99.7	19.89	< 0.0001
		36	35.1	27–42	0.0	0–0	100.0	21.75	< 0.0001

^a^Ticks supplied by Ecto Services, Inc. Colony originated from field-collected ticks in Stillwater, OK, in 2011 and was last supplemented with wild-caught ticks from Kittrell, NC, in 2019, ~9 months prior to study start

^b^Ticks supplied by Bertek, Inc. Colony originated from ticks field-collected in Greenbrier, AR, in 2009 and was last supplemented with wild-caught ticks from Greenbrier, AR, in 2020, ~2 months prior to study start

^c^Ticks supplied by Oklahoma State University. Ticks were field-collected in Payne County, OK, between 19 March and 4 June 2021, starting ~2 months prior to study start

^d^Ticks supplied by Ecto Services, Inc. Colony originated from ticks field-collected in Stillwater, OK, in 2011 and was last supplemented with wild-caught ticks from Kittrell and Dabney, NC, in 2021 ~13 months prior to study start

^e^First infestation on day −2. Subsequent infestations on day 5, 12, 19, 26, and 33. Tick counts were conducted at 72 h post-treatment and after weekly re-infestation

^f^Treatment with placebo or Revolution® Plus (minimum dosage 1.0 mg/kg sarolaner and 6 mg/kg selamectin) occurred on day 0

^g^Nine cats were included in this efficacy analysis; one cat was removed from study on day 33

### Animals

An identical study design was used in each of the four studies. There were two treatments per study (*n* = 10 per treatment): placebo-treated (control) and Revolution® Plus-treated. Cats were uniquely identified with alphanumeric ear tattoos, ranged in age from 7 to 92 months, and weighed between 2.3 and 6.4 kg at study initiation across all studies. Cats were examined by a veterinarian, determined to be in good health, and demonstrated the ability to maintain adequate tick infestations prior to treatment. In addition, each cat was observed twice daily for general health for the duration of the study. The cats were fed a commercial feline diet and had access to fresh water ad libitum throughout the study. Cats were housed individually in indoor enclosures in such a way that no physical contact between cats was possible throughout the study. To prevent cross-contamination after treatment administration, clean clothing and equipment were used for handling each cat, and personnel changed their clothing between cats.

### Tick strains and host suitability infestations

Ticks were obtained from three separate laboratory-maintained tick colonies. These colonies were originally established using ticks collected from the field from three different geographical areas of the USA and had wild-caught ticks introduced into the colony within the 3 years prior to the start of each study. The laboratories and origins of the tick strains used in each study are provided in Table 1. Individual vials of ticks for infestation were prepared for each cat prior to each infestation. In addition, prior to each infestation, three vials of ticks were randomly examined to check that the ticks had been correctly sorted according to number, sex ratio, viability, and unfed status. Tick host suitability was determined prior to inclusion of the cats in the study and within 5 days of treatment on day 0. For each study, between 24 and 30 cats were examined to verify that they were free of ticks before they were infested with 50 (± 5) viable, unfed *A. americanum* at a 1:1 sex ratio. After 72 h, live, attached ticks were counted and removed. The 20 cats with the highest tick counts were selected for each study.

### Experimental design

Day 0 was defined as the day the cats received treatment. Cats were allocated to treatments and cages according to a randomized complete block design, with blocks based on host-suitability tick counts. Within each block, each cat was randomly allocated to a treatment (Revolution® Plus or placebo), and two cats within each block were randomized to adjacent enclosures.

### Treatment

Cats were weighed, moved to their allocated housing, and infested with ticks on day −2. Either Revolution® Plus at the minimum dose (6.0 mg/kg body weight selamectin + 1.0 mg/kg body weight sarolaner) or placebo was administered on day 0. The dose of Revolution® Plus or placebo was rounded up to the nearest 0.1 mL as follows: the day −2 body weight in kg was multiplied by 0.1. When the first digit dropped was greater than zero, the preceding digit was increased by one (e.g., if the calculated dose was 0.31 or 0.39 mL, the cat received 0.4 mL).

The placebo was a vehicle control which contained the same inert formulation ingredients as Revolution® Plus but did not include the active ingredients, selamectin or sarolaner. Both the placebo and Revolution® Plus were applied to the respective allotted cats according to the label instructions, namely, directly to the skin at the base of the neck, cranial to the scapulae. All cats were observed immediately following dosing for potential adverse events associated with treatment and were observed again for general health and any potential reaction to treatment at 1, 3, 6, and 24 h after treatment. Evaluations of the application site were conducted at approximately 1, 3, 6, and 24 h after treatment and again at 3 and 5 days after treatment.

### Tick counts

Each cat was examined on day 3 (72 h after treatment) to remove and count all ticks remaining following infestation on day −2. In all studies, cats were re-infested with approximately 50 ticks on days 5, 12, 19, 26, and 33. Cats were examined, and ticks counted and removed 72 h after each re-infestation (days 8, 15, 22, 29, and 36). To facilitate infestation and enhance tick attachment, cats were lightly sedated in studies 2, 3, and 4 for each infestation. Cats were fitted with Elizabethan collars (all four studies) during the period from infestation on day −2 to treatment on day 0. On day 0, the collars were removed prior to treatment administration in order to minimize interference with the treatment application site. Except in study 1, the collars were replaced once the treatments had dried, approximately 24 h later, and subsequently removed on day 3 just prior to tick counts. For study 1, collars were removed on day 0 and were not replaced after treatment. In all studies, cats were fitted with collars at each subsequent infestation and removed at the next counting.

Tick counts were conducted by trained, masked personnel. The randomization plan, which allotted cats to cages also defined the sequence in which the cats were examined and all ticks were removed and counted. Each cat was initially examined to identify readily visible ticks by pushing the cat’s fur against its natural nap. Then an extra-fine-tooth comb was used to comb the cat thoroughly and identify any previously missed ticks. Cats were examined for a minimum of 10 min, and if any tick was encountered in the final minute, the combing was extended in 1-min increments until no additional ticks were encountered. All identified ticks were removed and examined to assess viability and the numbers of live and dead ticks were recorded. Viability of the ticks was assessed by gently blowing on them to expose them to CO_2_ or by applying tactile stimulus and observing any movement of the legs. Only live ticks (comprising live attached and live free) were used in the effectiveness calculations.

### Statistical analysis

The experimental unit for all studies was the individual cat, and the primary endpoint was live (attached and free) tick counts. Using the PROC MIXED procedure (SAS 9.4, SAS Institute Inc., Cary, NC), counts were analyzed using a mixed linear model for repeated measurements with treatment group, time point, and treatment by time point interaction as fixed effects; and block, block by treatment interaction (animal term), and error as random effects. Contrasts were used to compare treatment groups at each time point. Testing was two-sided at the significance level *α* = 0.05. Percentage efficacy was calculated from least squares means using Abbott’s formula:

$$\% \,{\text{reduction}}\,{\text{ = }}\,{\text{100}}\, \times \,\,\frac{{{\text{mean count }}({\text{placebo}})\,{\text{ - mean count }}({\text{treated}})}}{{{\text{mean count }}({\text{placebo}})}}$$

## Results

Cats in the placebo-treated groups in all four studies maintained adequate tick infestations for the study duration (“adequate” being defined as 12 or more of the ticks applied at infestation present on at least six of the ten placebo-treated cats on count days), with live (attached and free) ticks collected from all cats at all counts (Table 1). For most tick count days, at least 12–13 ticks, or a quarter of the ticks used per cat per infestation, were recovered from each cat. Across the four studies, least squares mean live (attached and free) *A. americanum* ticks recovered from cats in the control groups were between 17.4 and 36.4 ticks per cat and reflected between 34.8% to 72.8% of the number of ticks applied, respectively.

In study 1, Revolution® Plus provided 89.7% efficacy against existing *A. americanum* infestations within 72 h of treatment (day 3). Following weekly re-infestations, efficacy in this study was > 99.7% for 29 days after treatment and was 88.7% on day 36.

In study 2, Revolution® Plus provided 93.3% efficacy against existing *A. americanum* infestations within 72 h of treatment (day 3). Following weekly re-infestations, efficacy in this study was > 98.7% through day 36 after treatment.

In study 3, Revolution® Plus provided 88.5% efficacy against existing *A. americanum* infestations within 72 h of treatment (day 3). Following weekly re-infestations, efficacy in this study was 100% through day 36 after treatment.

In study 4, Revolution® Plus provided 98.4% efficacy against existing *A. americanum* infestations within 72 h post-treatment (day 3). Following weekly re-infestations, efficacy in this study was > 99.7% through day 36 after treatment.

Cats treated with Revolution® Plus had significantly lower least squares mean live tick counts than placebo-treated cats at all time points (*P* < 0.0001 for all tick counts).

### Safety

Abnormal health observations were recorded for a total of 19 cats from all studies, and none were deemed to be associated with treatment. Events recorded were mild reactions to tick infestation, including localized irritation and lesions owing to tick bite and mild hair loss (17 cats: 9 placebo-treated and 8 Revolution® Plus-treated cats), a single episode of vomiting (1 Revolution® Plus-treated cat), soft stool (1 Revolution® Plus-treated cat), and a minor facial abrasion (1 Revolution® Plus-treated cat). The authors determined that only the vomiting and soft stool, which were single incidents and were mild and self-limiting, may have been related to product administration.

## Discussion

The four studies confirmed the high and consistent efficacy of Revolution® Plus in treating existing infestations and in controlling re-infestations of *A. americanum* ticks at the minimum label dosage. A single topical dose of Revolution® Plus administered 2 days after tick infestation resulted in an efficacy of 92.5% (range: 88.5–98.4%) against existing *A. americanum* infestations within 72 h of product administration. In all studies, Revolution® Plus continued to reduce live tick numbers by > 90% following re-infestations for at least 29 days (*P* < 0.0001).

These data show that Revolution® Plus provides cats with highly effective treatment of existing infestations with *A. americanum* and also provides robust protection from re-infestation for a month after a single treatment.

The geographic range of the lone star tick in the USA has recently expanded into previously naive areas of North America, caused by many factors including warmer temperatures, human modification of the environment, and increased movement of human and animal hosts from endemic to nonendemic areas. *Amblyomma americanum* has now appeared in several new regions of the country [15, 16]. Along with the spatial shifts of the ticks, the prevalence of tick-borne diseases in animals also appears to be shifting, with some pathogens appearing to be re-emerging and others being reported in new geographic locales and host populations [17, 18]. For example, *C. felis* is a tick-transmitted protozoan, which causes bobcat fever, an emerging disease in the USA. Originally identified in the southeastern USA, *C. felis* is now moving northward along with the vector tick, *A. americanum* [9]. While domestic cats infected with *C. felis* can be treated with supportive care and antiparasitic drugs, between 40% and 90% of affected cats do not survive, even with aggressive and early treatment [19–21].

Transmission of *C. felis* to cats relies on a bite from *A. americanum* and occurs between approximately 36 h and 48 h post-tick infestation [22]. Revolution® Plus has been shown to block the transmission of *C. felis* to cats by killing infected *A. americanum* vector ticks in induced infestations [23]. In a previous study, three monthly doses of Revolution® Plus or the vehicle control (placebo) were administered to cats [23]. The cats were infested twice with adult *A. americanum* ticks. For the first infestation, which was performed 4 days after the first treatment using ticks not infected with *C. felis*, efficacy was assessed at 48 h and 72 h post-infestation and found to be 27.1% and 90.4%, respectively. The second infestation was done 4 days following the third treatment, this time using adult *A. americanum* ticks that had been acquisition-fed as nymphs on two *C. felis*-infected donor cats. Efficacy of the product assessed at 48 h and 72 h post-infestation was 56.4% and 94.7%, respectively. Seven of the eight placebo-treated cats became polymerase chain reaction (PCR)-positive for *C. felis* following infestation, while none of the eight Revolution® Plus-treated cats became positive. Given that transmission of *C. felis* may occur as early as 36 h post-infestation, while efficacy of the product at 48 h was only 56.4%, it was surprising that none of the Revolution® Plus-treated cats tested positive for the pathogen following infestation. The authors of the study hypothesized that slow onset of attachment by the ticks may have played a role here such that when ticks did attach and feed, they fed for < 36 h before being killed. Consequently, the window of opportunity to transmit the pathogen was effectively diminished.

Another observation made in this study [24] is that the efficacy at both 48 h and 72 h post-infestation improved following the third treatment compared with the first treatment. Prescribing information for Revolution® Plus states that sarolaner accumulates in the body with successive treatments such that the *C*_max_ plateaus after the third dose of sarolaner [24]. This likely explains the improved efficacy following the third treatment in the study [24]. While the studies reported in the present article did not focus on speed of kill, it is important to note that the mean efficacy of Revolution® Plus was > 90% at the first post-treatment evaluations. This efficacy at 72 h post-treatment is likely to increase with repeated treatments until three monthly treatments have been given.

Post-treatment evaluations over the course of 1 month showed efficacy > 99.1%, suggesting monthly administration of Revolution® Plus effectively protects cats from recurring tick infestations and will likely reduce the transmission of tick-borne pathogens.

## Conclusions

A single topical dose of Revolution® Plus administered at the minimum label dosage of 1 mg/kg sarolaner and 6 mg/kg selamectin topically provided a mean efficacy of > 90% against existing infestations of *Amblyomma americanum* ticks within 72 h of administration, and at least 99.1% efficacy 72 h after subsequent weekly infestations for 1 month. At all planned observations, Revolution® Plus-treated cats had significantly (*P* < 0.0001) lower live tick numbers compared with placebo-treated controls for at least a month.

Revolution® Plus provides owners and veterinarians with a highly effective means of treating and controlling infestations of *A. americanum* (lone star ticks) on cats for 1 month following a single topical application.

## Supplementary information

Additional file 1.

## Data Availability

‘Data supporting the main conclusions of this study are included in the manuscript.
