# Supplementary material for: Efficacy of Revolution® Plus (selamectin plus sarolaner) against Amblyomma americanum (lone star ticks) in cats
Source: Parasit Vectors. 2025 Aug 1;18:318. doi: 10.1186/s13071-025-06962-1 (PMC12315200; doi:10.1186/s13071-025-06962-1)

Efficacy of Revolution® Plus (RP) (6.0 mg/kg selamectin plus 1.0 mg/kg sarolaner) against *Amblyomma americanum* (lone star ticks) in cats

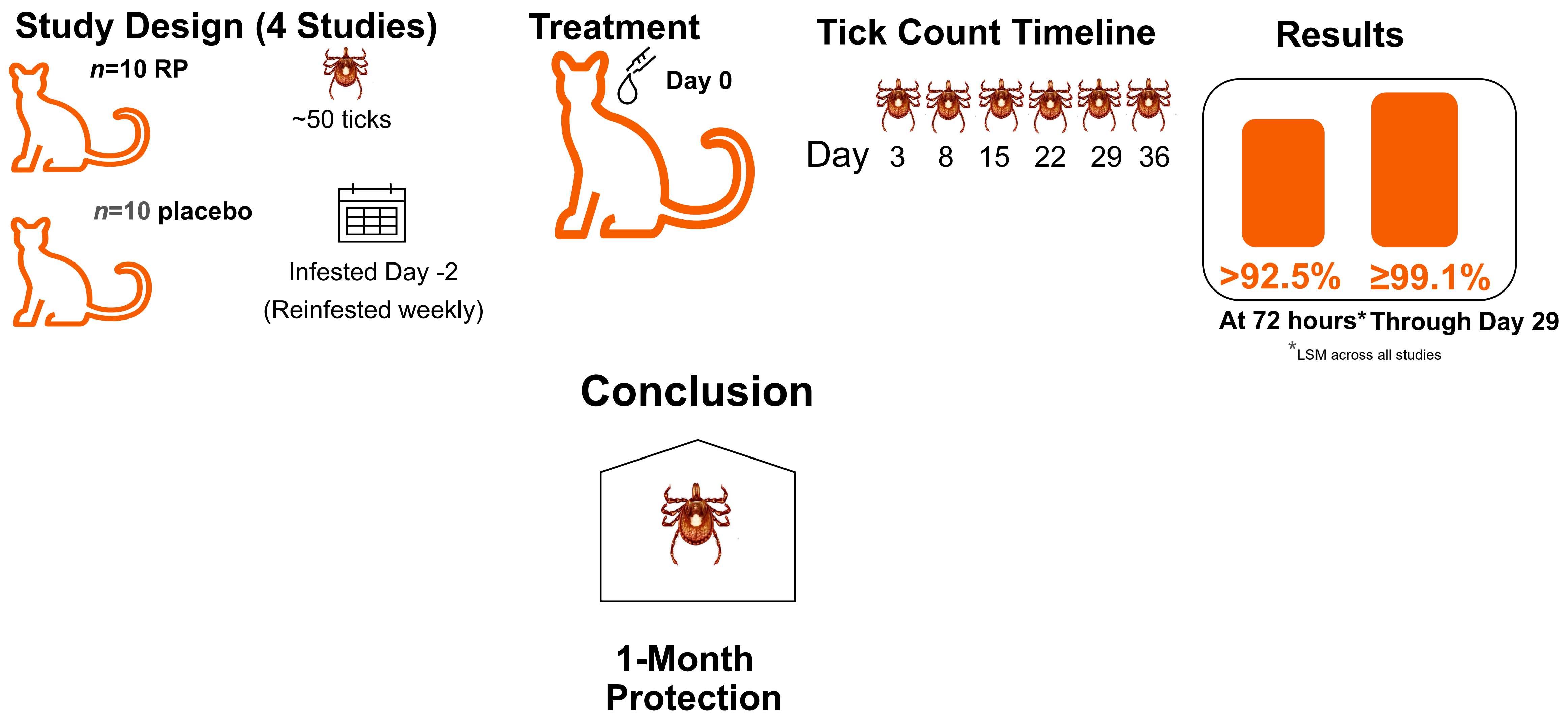

Supplement: Supplementary file 1 — Additional file 1. [file 13071_2025_6962_MOESM1_ESM.pdf]
